# Design Method of a Wide-Field, Dual-Slit, Low-Distortion, and High-Sensitivity Hyperspectral Imager

**DOI:** 10.3390/s25206478

**Published:** 2025-10-20

**Authors:** Xijie Li, Siyuan Li, Zhinan Zhang, Xiangpeng Feng, Zhong Shen, Xin Lu, Ming Gao

**Affiliations:** 1 School of Opto-Electronical Engineering, Xi’an Technological University, Xi’an 710021, China; lixijie@opt.ac.cn; 2 Shaanxi Key Laboratory of Optical Remote Sensing and Intelligent Information Processing, Xi’an Institute of Optics and Precision Mechanics, Chinese Academy of Sciences, Xi’an 710119, China; zhangzhinan@opt.ac.cn (Z.Z.); fengxiangpeng@opt.ac.cn (X.F.); shenzhong@opt.ac.cn (Z.S.);

**Keywords:** Offner hyperspectral imager, curved prism, spectral lateral deviation, spectral keystone, RSS discrete calculation

## Abstract

To increase target acquisition probability and the signal-to-noise ratio (SNR) of hyperspectral images, this paper presents a wide-field, dual-slit, low-distortion, and high-sensitivity Offner hyperspectral imager, with a wavelength range of 0.4 μm to 0.9 μm, a numerical aperture of 0.15, and a slit length of 73 mm. To avoid signal aliasing, the space between the dual slits is 2.4 mm, increasing the SNR by 1.4 times after dual-slit image fusion. Furthermore, to achieve the required registration accuracy of dual-slit images, the spectral performance of the hyperspectral imager is critical. Thus, we compensate and correct the spectral performance and dispersion nonlinearity of the hyperspectral imager by taking advantages of the material properties and tilt eccentricity of a low-dispersion internal reflection curved prism and high-dispersion double-pass curved prisms. To meet the final operation requirements, the tilt of the internal reflection curved prism is used as a compensator. Using the modulation transfer function (MTF) as the evaluation criterion, an inverse sensitivity analysis confirmed that the compensator is a highly sensitive component. Additionally, the root mean square standard deviation (RSS) discrete calculation method was adopted to assess the influence of actual assembly tolerance on spectral performance. The test results demonstrate that the hyperspectral imager meets the registration accuracy requirements of dual-slit images, with an MTF better than 0.4. Furthermore, the spectral smile and spectral keystone of the dual-slit images are both less than or equal to 0.3 pixels.

## 1. Introduction

Currently, imaging spectrometers are widely used in various fields, including remote sensing, biomedicine, chemical detection, mineral exploration, environmental monitoring, missile interception, explosion analysis, and combustion diagnostics [1,2,3,4,5]. Pushbroom imaging spectrometers have two dimensions, capturing spatial data parallel to the slit and acquiring the corresponding spectral information perpendicular to the slit. Spatial information along the orbital direction is acquired through a satellite’s movement along its orbit over time, ultimately generating a three-dimensional data cube containing spatial and spectral information [6,7]. Unlike traditional planar dispersion prisms, curved prism spectral imaging technology can be applied to non-parallel optical paths, with advantages, like compact structure, low nonlinear dispersion, and aberration correction, significantly improving imaging quality and overall system performance [8]. On-orbit high-resolution curved prism hyperspectral imagers include the CHRIS system, the STSAT3 system, the EnMap system, and micro-nano spectrometers [9,10,11,12]. However, although curved prism spectrometers have advantages such as low spectral keystone and high spectral resolution, their processing and tuning are more difficult to work with than those of traditional grating dispersion spectrometers owing to their non-coaxial features. Rational allocation of processing and alignment tolerances for curved prisms ensures instruments have excellent spectral performance and imaging quality and are thus suitable for engineering.

The impact of optical system manufacturing and assembly errors on system imaging performance can be determined through traditional Monte Carlo tolerance analysis. However, this tolerance analysis and evaluation method fails to provide impact analysis on indicators, like system distortion magnification, telecentricity, spectral smile, and spectral keystone. L.B. Moore et al. proposed an evaluation method for imaging spectrometer that simultaneously satisfies five variables, namely, along-track response functions, cross-track response functions, spectral response functions, spectral centroid uniformity, and spatial centroid uniformity [13]. This method quantifies spectral smile and keystone, enabling rapid and semi-automated assessment from the design stage to processing, assembly, and the orbital cycle. H. Ku et al. designed an inverse sensitivity evaluation method for traditional Offner imaging spectrometers, assessing tolerance budgets for each component in practical production [14]. By using the tilt and eccentricity of the secondary mirror as compensators, they were able to perform a multi- performance tolerance analysis of the system’s MTF, diapoint, spectral smile, and spectral keystone. L. Feng et al. designed a broadband Offner imaging spectrometer covering 0.4 μm–2.5 μm, ensuring the spectral performance of the system through MTF Monte Carlo analysis [15].

In this study, the spectral performance of a hyperspectral imager was analyzed by using the MTF evaluation method. The tilt of its curved prism was adopted as the compensator, verifying its sensitivity through inverse sensitivity analysis. Further, the RSS discrete calculation method was used to evaluate processing and assembly errors, ultimately achieving excellent imaging and spectral performance.

## 2. Impact of Processing and Assembly Errors on Spectral Performance

**Figure 1:**
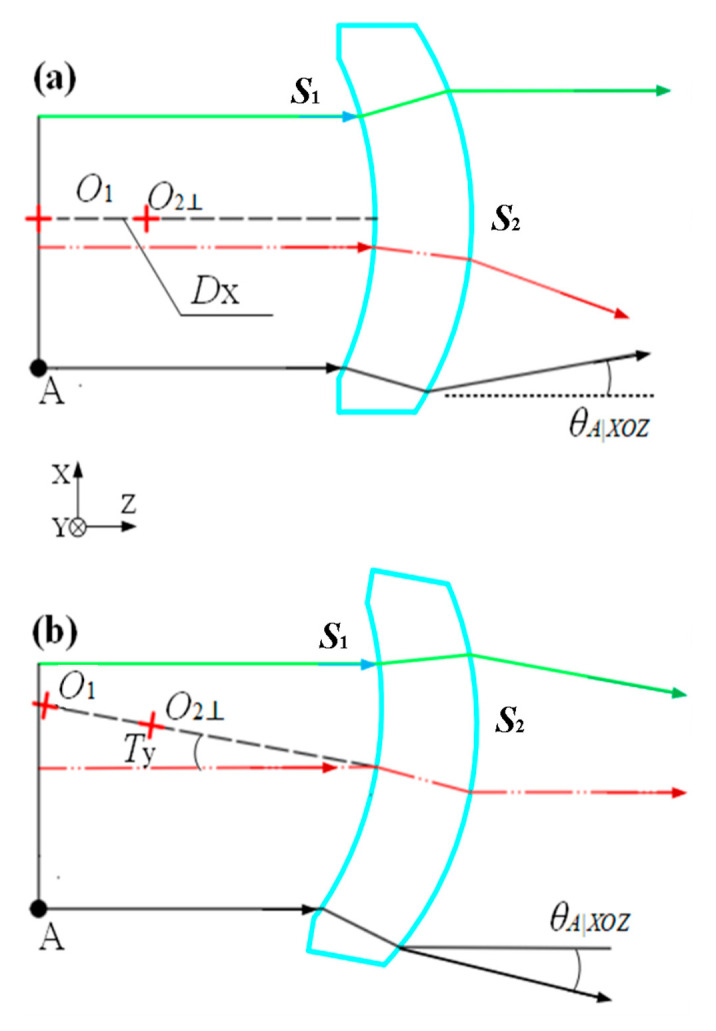
Schematic diagram of assembly error of the curved prism. (**a**) Assembly decentration error; (**b**) Assembly tilt error.

Processing and assembly errors of the curved prism can cause variations in the incident angle and height of emitted light rays from each slit point on its surface. Figure 1 illustrates the assembly errors in the curved prism under study through geometric ray tracing.

Here, *D_x_* represents the displacement deviation of the curved prism along the *x*-axis, and *T_y_* denotes the rotational deviation along the *y*-axis.

Spectral smile can be authentically quantified using spectral calibration data, and corrected through data processing and differential fitting. A spectral keystone can be created through polychromatic light star point centroid fitting, yet correcting it is very difficult [16]. Hence, this section focuses on analyzing the impact of the curved prism on the spectral keystone. Ideally, the light refraction transfer matrix of the curved prism in the *x*-axis direction can be expressed as follows:

(1) $$N_{2}=M_{S_{2}}\cdot M_{t}\cdot M_{S_{1}}\cdot N_{1}$$

(2) $$M_{sq}=\left[\begin{array}{cc} 1 & 0\\ \frac{n^{\prime }-n}{r_{q}} & 1 \end{array}\right],\;q=1,2$$

(3) $$N_{q}=\left[\begin{array}{c} h_{q}\\ n_{q}\sin u_{q} \end{array}\right]$$

(4) $$M_{S_{2}\;|XOZ}=\left[\begin{array}{cc} 1 & -\frac{\left|P_{1}P_{2}\right|}{n_{prism}}\\ 0 & 1 \end{array}\right]\cdot M_{S_{1}|XOZ}$$

where *N*_2_ is the refraction matrix of the curved prism in the *x*-axis direction; *M_sq_* is the refraction matrix for a single spherical surface; *M_t_* is the transition matrix from *S*_1_ to *S*_2_ (with *S*_1_ and *S*_2_ indicated in the diagram); *n*′ is the refractive index of the medium on the refractive side; *n* is the refractive index of the medium on the incident side; *r_q_* is the radius of the *q*th spherical refractive surface; *n_q_* is the refractive index of the material of the qth spherical refractive surface; *n_q_* is the ray matrix; *M_S_*_2|*XOZ*_ is the transition matrix between the spherical surfaces along the *x*-axis; *M_S_*_1|*XOZ*_ is the refraction matrix of the spherical surface along the *y*-axis. *n* is the refractive index of the curved prism; *h_q_* is the distance from the spherical surface intersection point to the principal axis; and *u_q_* is the angle between the chief ray of the refractive surface and the principal axis of the spherical surface.

When processing and assembly errors occur in the curved prism, the incident light at slit point A deflects in the *XOZ* plane. The dispersion angle of the light at point A along the *X*-axis is derived from Equations (1)–(4), and the light deflection angle at point A in the *X*-axis direction is *θ_A_*_|*XOZ*_ [17,18,19].

(5) $$\left[\begin{array}{l} {h^{\prime }}_{Final}\\ n_{air}\sin(\theta_{A|XOZ})+T_{x} \end{array}\right]=\left[\begin{array}{cc} 1 & 0\\ \frac{n_{air}-n^{\prime }}{R_{2|XOZ}} & 1 \end{array}\right]\cdot \left[\begin{array}{cc} 1 & -\frac{(\left|P_{1}P_{2}\right|-\frac{D_{y}}{\tan\alpha })\cos T_{y}}{n_{prism}}\\ 0 & 1 \end{array}\right]\cdot \left[\begin{array}{c} {h^{\prime }}_{1}\\ n_{prism}\sin{U^{\prime }}_{1|XOZ} \end{array}\right]$$

(6) $$\left[\begin{array}{l} {h^{\prime }}_{1}\\ n_{prism}\sin{U^{\prime }}_{1|XOZ} \end{array}\right]=\left[\begin{array}{cc} 1 & 0\\ \frac{n^{\prime }-n_{air}}{R_{1|XOZ}} & 1 \end{array}\right]\cdot \left[\begin{array}{c} \frac{(-d+D_{x})\cos T_{z}}{\cos T_{x}}\\ n_{air}\sin T_{x} \end{array}\right]$$

where ${h^{\prime }}_{Final}$ is the distance from the intersection point of the emitted ray from the curved prism and the rear surface to the principal optical axis of the rear surface; *T_x_* is the tilt error of the curved prism around the *x*-axis; *D_y_* is the eccentric error in the *y*-axis direction; *T_z_* is the tilt error around the *Z*-axis; ${h^{\prime }}_{1}$ is the distance from *S*_1_ to the principal axis of *S*_1;_ and the angle between the refracted ray after passing through S_1_ and the principal axis of *S*_1_ is ${U^{\prime }}_{l丨XOZ}$.

## 3. Spectroscopic System Design

In the design process, the first step is to determine the design input of a hyperspectral imager, including wavelength, spectral resolution, etc.; the second step is to determine the initial structural configuration, including off-axis displacement, material, optical power, and air space. Finally, the design parameters of the system are determined as shown in Table 1.

**Figure 2:**
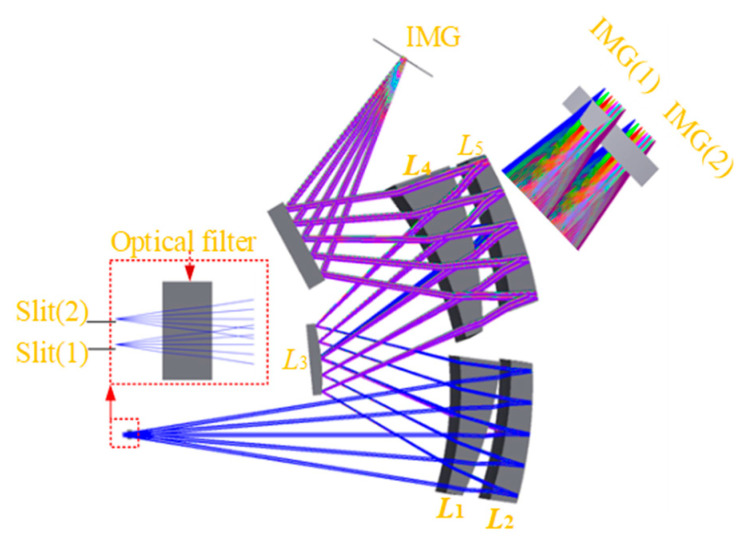
Optical path diagram of the spectroscopic system.

The initial configuration of the hyperspectral imager is an Offner concentric structure, achieving dispersion width through different off-axis displacement of curved prisms and the element tilt, and correcting dispersion non-uniformity through material matching. The final light path diagram of the system is illustrated in Figure 2, primarily consisting of four curved prisms and a secondary mirror, and the volume is 445 mm × 230 mm × 461 mm. The parameters of the optical elements are detailed in Table 2.

### 3.1. System Design

**Figure 3:**
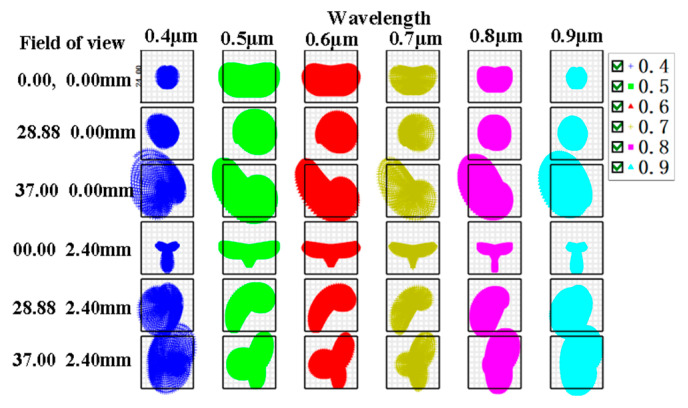
Matrix spot diagrams of the system at different sampling wavelengths and slit lengths.

**Figure 4:**
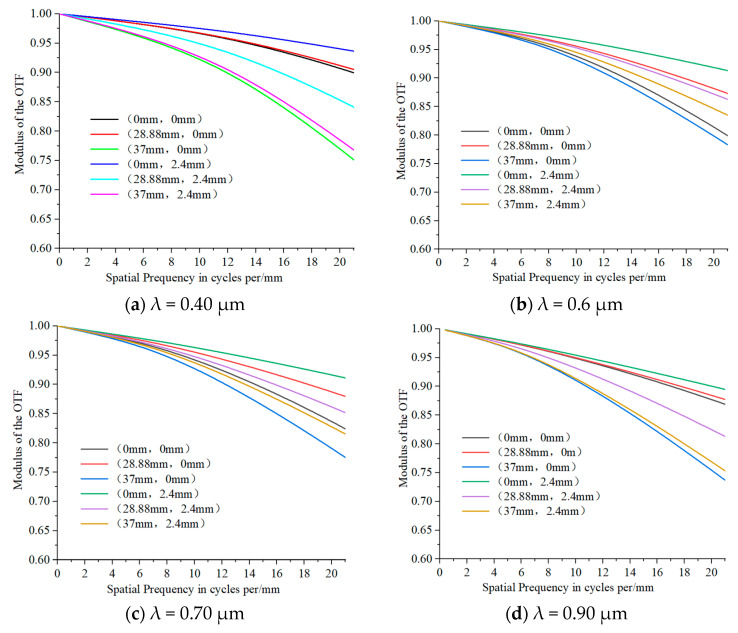
MTF of the system at different wavelengths.

The system’s matrix spot diagrams of the sampling wavelengths of 0.4 μm, 0.5 μm, 0.6 μm, 0.7 μm, 0.8 μm, and 0.9 μm at the slit lengths of (0 mm, 0 mm), (28.88 mm, 0 mm), (37 mm, 0 mm), (0 mm, 2.4 mm), (28.88 mm, 2.4 mm), and (37 mm, 2.4 mm) are shown in Figure 3, illustrating that the system’s matrix spot diagrams at different sampling wavelengths and slit lengths are within a single pixel (24 μm). The system’s MTF of the sampling wavelengths of 0.4 μm, 0.6 μm, 0.7 μm, and 0.9 μm at various slit lengths are presented in Figure 4, observing that the system’s MTF of different sampling wavelengths at the entire slit lengths exceeds 0.75 at the Nyquist frequency of 21 lp/mm across. In conclusion, the imaging quality of the system is excellent.

The spectral smile and spectral keystone affect the detection accuracy and recognition accuracy of spectroscopic instruments, respectively, thus correcting them is very important.

**Figure 5:**
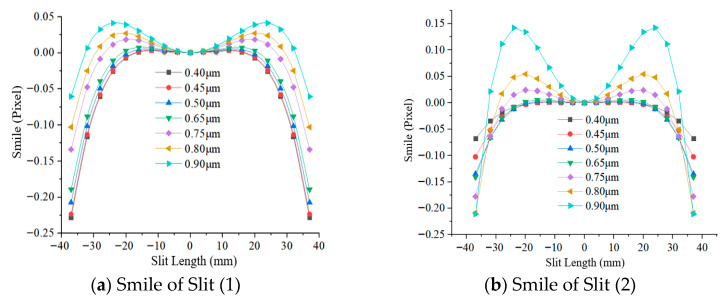
Spectral smile of the system at different wavelengths.

**Figure 6:**
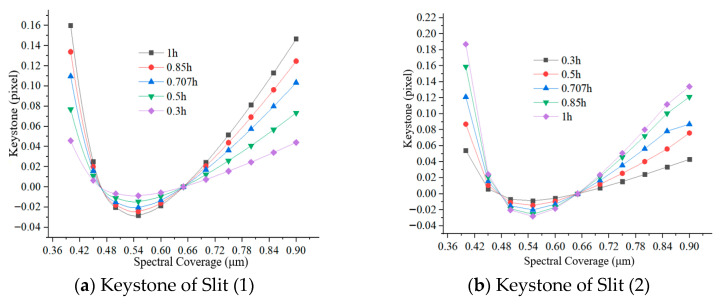
Spectral keystone curve of the system at different FOVs.

**Figure 7:**
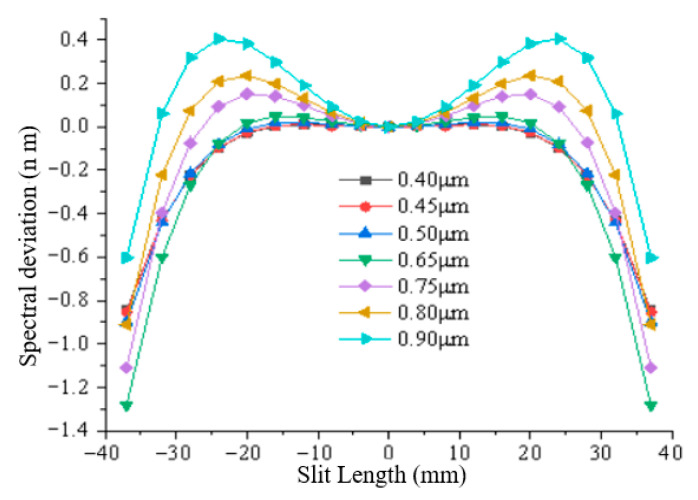
Spectral lateral deviation curve of the system at different wavelengths.

Taking the center of the slit as the reference, the spectral difference between the different slit lengths at the same sampling wavelength and the reference are spectral smile. As shown in Figure 5, the spectral smiles of the system were fitted at sampling wavelengths of 0.40 μm, 0.45 μm, 0.50 μm, 0.65 μm, 0.75 μm, 0.80 μm, and 0.90 μm, indicating that the spectral smile of Silt (1) and Silt (2) at different sampling wavelengths are both less than 0.3 pixels. Spectral keystone fitting is based on the sampling center wavelength as the reference, the spectral difference between the different sampling wavelengths at the same FOV and the reference are spectral keystone. The system’s spectral keystones of different sampling wavelengths at 0.3 h, 0.5 h, 0.707 h, 0.85 h and 1 h are shown in Figure 6, indicating that the spectral keystones of Sill (1) and Sill (2) at different FOVs are both less than 0.24 pixels. The spectral lateral deviation fitting curve of the system, as shown in Figure 7, is less than 2 nm. Therefore, the spectral smile and spectral keystone can be corrected without data processing.

### 3.2. System Assembly Tolerance Analysis

Using MTF as the evaluation criterion, an inverse sensitivity Monte Carlo analysis method was employed to analyze the preset tolerances of the processing and assembly of the system. The assembly tolerances of Prisms 2 and 5 served as compensators. The preset tolerance values of the system are listed in Table 3 and Table 4.

**Figure 8:**
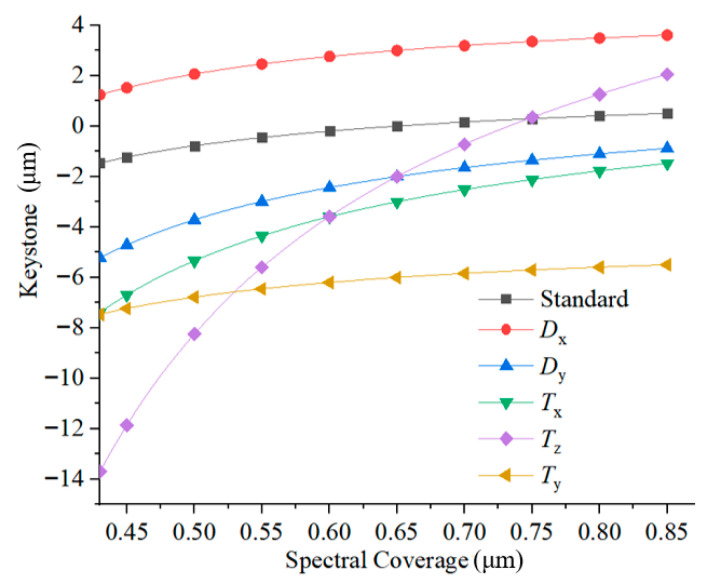
Influence of error perturbation on spectral keystone at different wavelengths.

Since the elements can compensate for spectral performance, using Monte Carlo methods to analyze the spectral performance will lead to inaccurate results. The eccentricity of the spectral keystone and tilt adjustment error function includes five variables. The sensitivity of each variable to the spectral keystone is shown in Figure 8, suggesting that the spectral keystone is particularly sensitive to the *T_z_* and *T_x_* tolerance terms.

**Figure 9:**
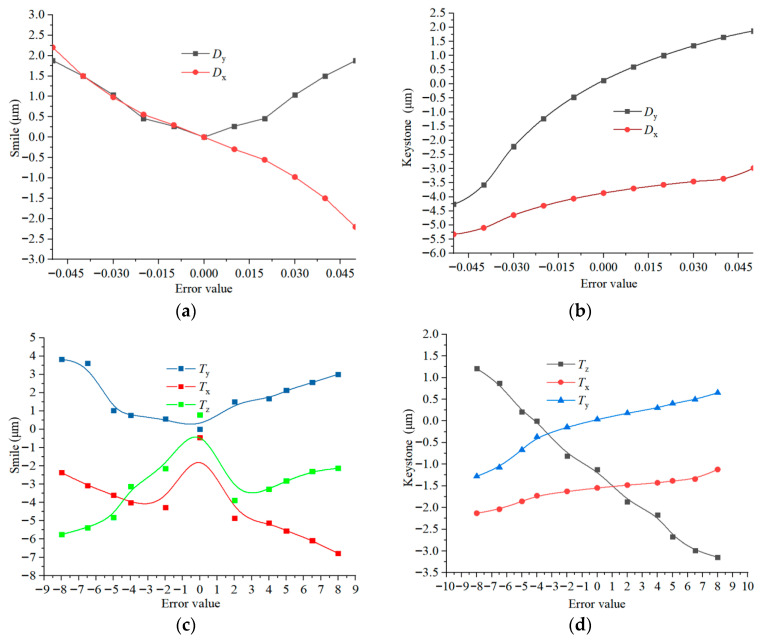
Spectral smile and spectral keystone under tilt and decentration errors. (**a**,**b**) present the influences of tilt on spectral smile and spectral keystone, respectively; (**c**,**d**) present the influences of decentration on spectral smile and spectral keystone, respectively.

The error ranges for the decentration (*D_x_*, *D_y_*) of the compensation mirror group are set from −0.05 mm to 0.05 mm, and the tilt errors (*T_x_*, *T_y_*, *T_z_*) are set from −8′ to 8′. Taking a wavelength of 0.6328 μm as an example, the sensitivity of the system within this error range is analyzed (Figure 9). The figure indicates that *T_z_* is the most sensitive to the spectral keystone, *D_x_* is the most sensitive to the spectral smile, and individual alignment errors can mutually compensate for each other.

To validate the tolerance sensitivity of the system’s spectral performance, the extremum method was adopted to obtain discrete data for the positive increment of the spectral performance caused by the assembly tolerances. Then, the RSS method was adopted to calculate the maximum positive increment for each discrete datapoint on spectral performance, as shown in the following equation:

(7) $$\mathrm{RSS}=\sqrt{\sum_{j=1}^{N}x_{j}^{2}}$$

From Table 5, it can be concluded that the limit increment of the spectral smile caused by the element eccentricity and tilt assembly tolerances is 1.502 µm, and the maximum design residual of the spectral smile is 5.546 µm. Therefore, the final maximum spectral smile is 7.048 µm, which is less than 7.2 µm (0.3 pixels).

## 4. Performance Test

**Figure 10:**
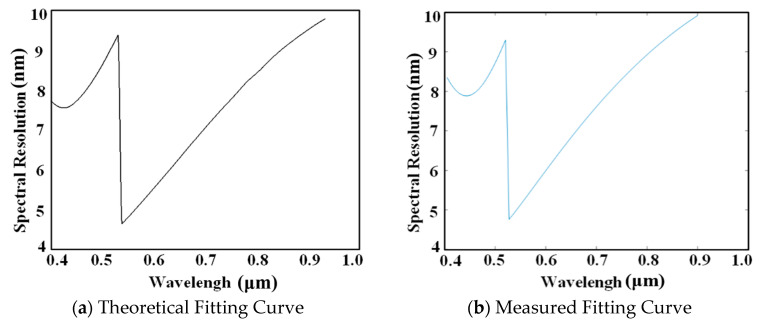
The fitting curves of the spectral resolution.

The theoretical fitting curve of the spectral resolution is shown in Figure 10a. The theoretical average sampling resolution of the system is 6.0102 nm, with a theoretical maximum sampling resolution of 9.7781 nm. A monochrometer was used to obtain slit images at the center pixel of each channel. The centroids of these slit images underwent difference fitting, yielding the system’s sampling resolution as shown in Figure 10b. The fitted results show an average sampling resolution of 7.5314 nm and a maximum sampling resolution of 9.9226 nm, aligning closely with the theoretical fitting curve. The test results fully confirm the effectiveness of the off-axis lens group in correcting the spectral smile.

**Figure 11:**
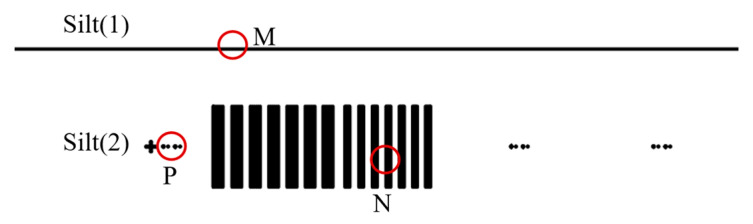
Test version pattern.

For such systems with complex spatial positions and intricate curved prism processing and assembly, the indicators of the dual slits should be tested. In order to effectively test indicators of the system, a test slit was manufactured, as shown in Figure 11. Slit (1) is the slit image, which can monitor the parallelism between the slit and the CCD and test the spectral smile. Slit (2) are star points and stripe plate that can test the magnification, MTF and spectral keystone of the system. The test striped plate is illustrated in Figure 11. When all indicators meet the requirements, the test slit is rotated 180° for comparative test in both states, ensuring that all indicators of Slit (1) and Slit (2) satisfy the requirements.

**Figure 12:**
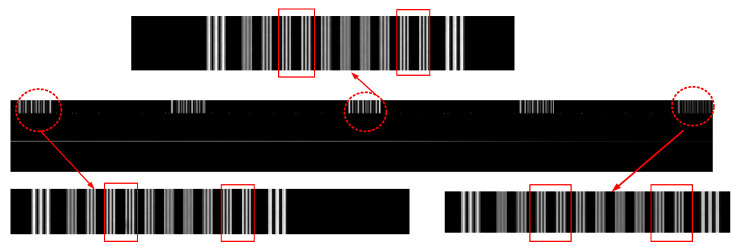
Principle schematic diagram of the MTF test.

The laser at different wavelengths were used to uniformly illuminate the slit. The MTF of the system for different wavelengths at different FOVs were calculated according to Equation (8). The principle schematic diagram of the MTF test is shown in Figure 12. The calculation results of the MTF of the system at different fields of view under wavelengths of 405 nm, 635 nm, 780 nm, and 808 nm are presented in Table 6.

(8) $$\mathrm{MTF}\approx \frac{\pi }{4}\times (\frac{DN_{bright}-DN_{dark}}{DN_{bright}+DN_{dark}-2\times DN_{noise}})$$

**Figure 13:**
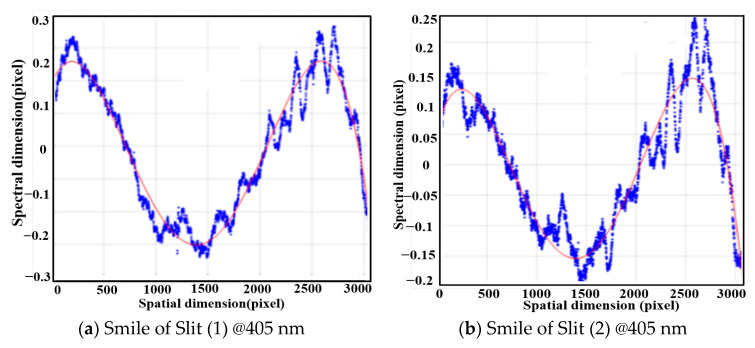

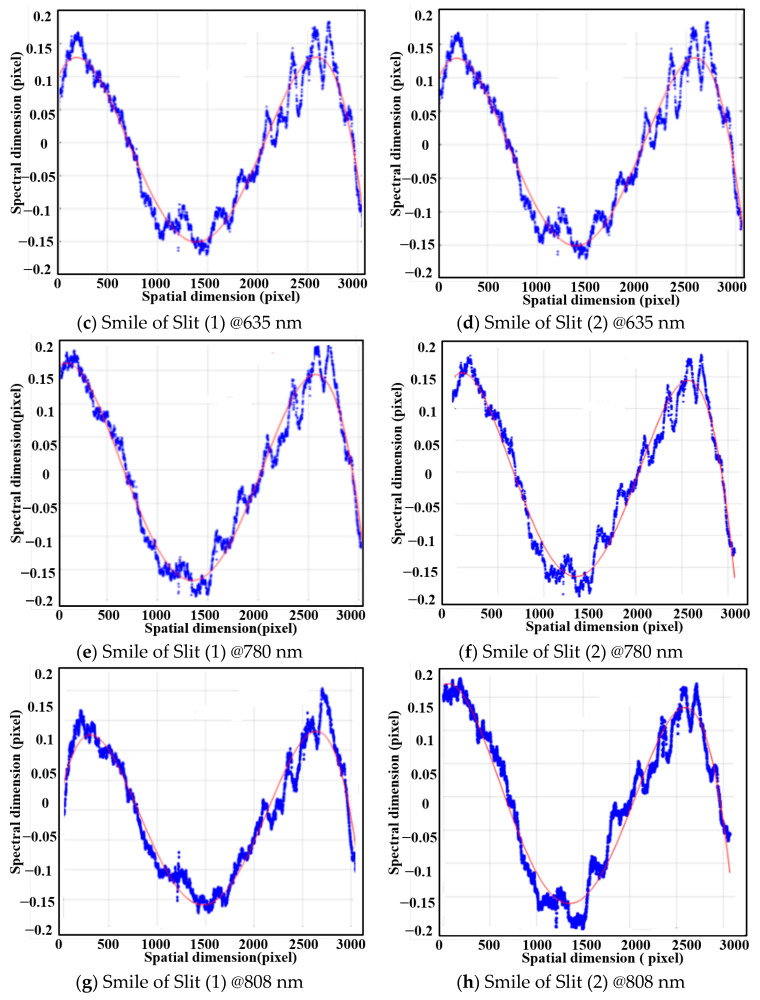
Measured fitting curves of spectral smile of the system at different wavelengths.

The 405 nm, 635 nm, 780 nm, and 808 nm lasers were used to fit and calculate the spectral smile at different wavelengths. The fitting curves are shown in Figure 13, and the corresponding spectral smile of 0.3669 pixel @405 nm, 0.227 pixel @ 635 nm, 0.3286 pixel @780 nm, and 0.1848 pixel @808 nm.

The spectral lateral deviation data of the system at different wavelengths are listed in Table 7.

**Figure 14:**
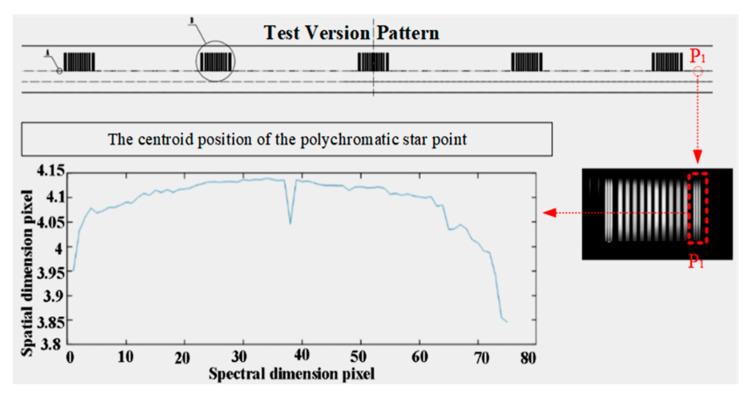
Test fitting curve of spectral keystone.

Continuous polychromatic spectra were attained through uniform polychromatic light illumination of star point images. The centroids of these spectra were fitted to calculate the keystone of the spectrometer. According to the test results in Figure 14, the maximum spectral keystone reaches 0.2451 pixels.

**Figure 15:**
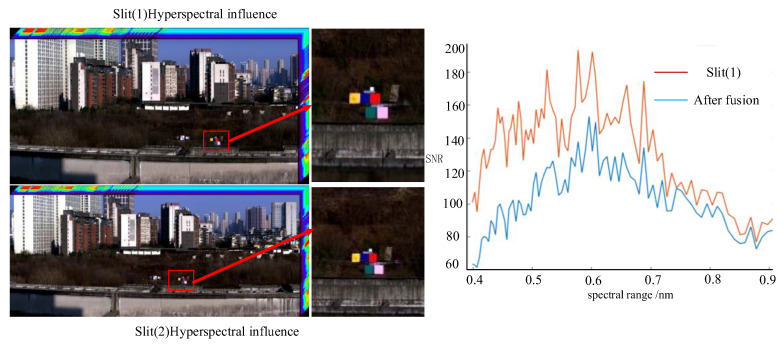
Field push-broom data cube of the hyperspectral imager.

In February 2025, a field push-broom experiment was conducted using the prototype. The double-slit data cube and SNR derived from push-broom scanning are illustrated in Figure 15. The image contours are clearly distinguishable, and the imaging quality is excellent. The SNR after the dual-slit fusion is 1.4 times that of the Slit (1) image.

## 5. Conclusions

This paper presents a wide-field, dual-slit, low-distortion, and high-sensitivity Offner hyperspectral imager. A sensitivity tolerance analysis method is proposed to improve the dual-slit image fusion precision of the hyperspectral imager and to acquire high-quality image data. The influence of actual assembly errors on spectral performance is evaluated through the Monte Carlo analysis and RSS methods. Finally, the prototype was tested, demonstrating that the SNR of the dual-slit image fusion is 1.4 times that of the single-slit image, significantly improving the detection sensitivity of the system. The test value of the spectral smile and spectral keystone closely align with the design value, effectively validating the feasibility of the inverse-sensitivity Monte Carlo analysis and RSS tolerance analysis methods.

## Figures and Tables

**Table 1:** System specification.

Parameters	Value
Wavelength	0.4 μm~0.9 μm
Spectral Resolution	≤10 nm
Width of Slit	24 μm
Length of Slit	73 mm
Pixel size	24 μm
Image format	3072 × 256
Relative aperture	0.15
Spectral band	68
lateral magnification	1:1
Spectral lateral deviation	≤3 nm
Spectral keystone	≤0.3 pixel
Object’s telecentricity	≤0.5°

**Table 2:** Optical parameters.

Type	Curvature /mm	Prism Thickness /mm	Air Space /mm	Optical Material	Prism Wedge Angle/°	Off-Axis/mm	Tilt /°
*L*_1_	−367.82	38	50.82	H-K9L	10.391	11.2	5.85
	−418.365						
*L*_2_	−426.95	26.5	149.7	H-TF8	2.256	5.1	8.98
	−449						
*L*_3_	−218.02	40	127.5	F_SILICA	-	80	−5.64
*L*_4_	−383.5	61.7	52	H-K9L	13.15	185.55	−26.05
	−415.323						
*L*_5_	−435.486	35.51	26	H-TF8	4.213	209.5	−16.83
	−460.724						

**Table 3:** The preset machining tolerance of the system.

Type	Δ*R*/mm	Δ*d*/mm	Δ*θy*/°	RMS @632.8 nm
*L*_1_	0.047	0.03	10.4°	1/45λ
*L*_2_	0.051	0.03	2.2°	1/45λ
*L*_3_	0.02	0.1	-	1/70λ
*L*_4_	0.036	0.03	13.06°	1/45λ
*L*_5_	0.041	0.03	3.91°	1/45λ

**Table 4:** The preset assembly tolerances of the system.

Relative Positional Deviation							Notes
**Type**	**Δ*x* (mm)**	**Δ*y* (mm)**	**Δ*z* (mm)**	**Δ*θx* (°)**	**Δ*θy* (°)**	**Δ*θz* (°)**	
*L*_1_	0.03	0.03	0.03	15″	15″	15″	
*L*_2_	0.03	0.03	0.03	15″	15″	15″	Compensator
*L*_3_	-	-	-	-	-	-	Reference
*L*_4_	0.03	0.03	0.03	10″	10″	15″	
*L*_5_	0.03	0.03	0.03	10″	10″	15″	Compensator

**Table 5:** The influence of actual assembly errors on spectral performance based on RSS method.

Type	Tilt Error						Eccentric Error			
	**ΔSmile (μm)**			**ΔKeystone (μm)**			**ΔSmile (μm)**		**ΔKeystone (μm)**	
	***θx***	***θy***	***θz***	***θx***	***θy***	***θz***	**Δ*x***	**Δ*y***	**Δ*x***	**Δ*y***
*L*_1_	0.115	0.126	0.415	0.02	0.05	0.08	0.045	0.04	0.024	0.003
*L*_2_	0.821	0.002	0.13	0.041	0.00112	0.1	0.005	0.351	0.003	0.005
*L*_4_	0.182	0.648	0.537	0.03	0.018	0.12	0.41	0.04	0.21	0.015
*L*_5_	0.4798	0.33	0.145	0.042	0.121	0.27	0.012	0.017	0.142	0.021
RSS	1.412			0.3548			0.544958		0.256	

**Table 6:** MTF calculation results for different wavelengths at different FOVs.

Type	Slit(1) MTF Average			Slit(2) MTF Average		
	**−1 h**	**0 h**	**1 h**	**−1 h**	**0 h**	**1 h**
405 nm	0.5279	0.5549	0.4467	0.521	0.505	0.489
635 nm	0.541	0.5611	0.5112	0.571	0.552	0.478
780 nm	0.504	0.545	0.481	0.578	0.567	0.459
808 nm	0569	0.563	0.487	0.584	0.508	0.487

**Table 7:** Measured data of spectral lateral deviation.

Number	Wavelength /nm	Smile /pixel	Spectral Resolution/nm	Spectral Lateral Deviation/nm
1	405 nm	0.3669	3.7 nm	1.36 nm
2	635 nm	0.2270	6.5 nm	1.48 nm
3	780 nm	0.3286	8.7 nm	2.86 nm
4	808 nm	0.1848	9.0 nm	1.66 nm

## Data Availability

The data presented in this study are included in the article.
